# Development of Evidence-Based Health Policy Documents in Developing Countries: A Case of Iran

**DOI:** 10.5539/gjhs.v6n3p27

**Published:** 2014-02-08

**Authors:** Mohammad Hasan Imani-Nasab, Hesam Seyedin, Reza Majdzadeh, Bahareh Yazdizadeh, Masoud Salehi

**Affiliations:** 1 Department of Health Services Management, School of Health Management and Information Sciences, Iran University of Medical Sciences, Tehran, Iran; 2 Health Management and Economics Research Center, School of Health Management and Information Sciences, Iran University of Medical Sciences, Tehran, Iran; 3 Knowledge Utilization Research Center, School of Public Health, Tehran University of Medical Sciences, Tehran, Iran; 4 Department of Epidemiology & Biostatistics, School of Public Health, Tehran University of Medical Sciences, Tehran, Iran; 5 Department of Statistics and Mathematics, School of Health Management and Information Sciences, Iran University of Medical Sciences, Tehran, Iran

**Keywords:** research utilization, policies, health policy, theory of planned behavior, Iran

## Abstract

**Background:**

Evidence-based policy documents that are well developed by senior civil servants and are timely available can reduce the barriers to evidence utilization by health policy makers. This study examined the barriers and facilitators in developing evidence-based health policy documents from the perspective of their producers in a developing country.

**Methods:**

In a qualitative study with a framework analysis approach, we conducted semi-structured interviews using purposive and snowball sampling. A qualitative analysis software (MAXQDA-10) was used to apply the codes and manage the data. This study was theory-based and the results were compared to exploratory studies about the factors influencing evidence-based health policymaking.

**Results:**

18 codes and three main themes of behavioral, normative, and control beliefs were identified. Factors that influence the development of evidence-based policy documents were identified by the participants: behavioral beliefs included quality of policy documents, use of resources, knowledge and innovation, being time-consuming and contextualization; normative beliefs included policy authorities, policymakers, policy administrators, and co-workers; and control beliefs included recruitment policy, performance management, empowerment, management stability, physical environment, access to evidence, policy making process, and effect of other factors.

**Conclusion:**

Most of the cited barriers to the development of evidence-based policy were related to control beliefs, i.e. barriers at the organizational and health system levels. This study identified the factors that influence the development of evidence-based policy documents based on the components of the theory of planned behavior. But in exploratory studies on evidence utilization by health policymakers, the identified factors were only related to control behaviors. This suggests that the theoretical approach may be preferable to the exploratory approach in identifying the barriers and facilitators of a behavior.

## 1. Introduction

Evidence is experienced or observed facts that support a conclusion. Research evidence is the most convincing type of evidence. It plays an important role in every stage of the policy making process—from setting agendas and developing policies to implementing and evaluating them (Dobrow, Goel, & Upshur, 2004). Evidence-based medicine approach was introduced in the 1990s (Claridge & Fabian, 2005). This approach entered the area of health policy soon and resulted in the development of various theories, frameworks, instruments, and processes (Lavis & Oxman, 2009; Orem et al., 2012; Packwood, 2002)

Publication of 2004 WHO report, the 58^th^ World Health Assembly, and Bamako Declarations encouraged health systems to incorporate research evidence into health policymaking (World Health Organization [WHO], 2004, 2005, 2008). Accordingly, some changes were made in Iran’s Health System (IHS). The most important ones were establishment of the National Institute of Health Research, Health Technology Assessment Office, and Health Policy Council in Iran’s Ministry of Health (IMoH). Concurrent with these developments, the High Council of Health and Food Security, the highest health policy authority at the government, announced that it accepts only evidence-based policy documents (EBPDs) on its agenda (Iran’s National Institute of Health Research, 2013; Iran’s Supreme Council of Health, and Food Security, 2013; Larijani, Delavari, Damari, Moghadam, & Majdzadeh, 2009; Majdzadeh, Nedjat, Fotouhi, & Malekafzali, 2009).

IHS uses research evidence in different health policy mechanisms for delivery, financing, governance, and implementation of health policies. However, its efforts are not systematic, comprehensive, and well institutionalized. For example, adding new services to the Iranian primary healthcare system requires adequate evidence, but there is no such commitment at the hospital level. Health technology assessment projects are needed to import major medical equipment to the country. Production or import of new drugs also requires enough evidence, but these efforts were not made for every major drug or medical equipment.

Integration of health services and medical education in IHS provides an opportunity to bridge the know-do gap (Majdzadeh, Yazdizadeh, Nedjat, Gholami, & Ahghari, 2012). For example, scientific committees consisting of scholars and administrative experts have been working in the technical offices of IMoH to provide scientific support for health policies. Similar to the most developing countries, the Iranian health policy system is centralized. IMoH is the main authority for health policymaking. The Fifth Economic, Social, and Cultural Development Plan (2009-2014) mandates the components of IHS to adhere to the governance of IMoH (Iran’s parliament [IP], 2009a). Medical universities act as IMoH executive arms. Recently, some efforts have been made to decentralize health policymaking. One example is the establishment of the board of trustees in medical universities (IP, 2009b).

### 1.1 Material Studied

Some studies have been carried out on the factors that influence evidence utilization by health policy makers. Lack of timely access to relevant evidence, use of jargon, limited time, poor search, critical appraisal, adaption skills, and lack of communication with researchers were identified as barriers to the use of evidence in health policy making (Albert, Fretheim, & Maïga, 2007; Bowen, Erickson, Martens, & Crockett, 2009; Campbell et al., 2009; Ellen et al., 2013; Hennink & Stephenson, 2005; Hyder et al., 2011; Jewell & Bero, 2008; Majdzadeh et al., 2012; Orem et al., 2012). Several strategies were suggested to address these barriers: access to databases that publish systematic reviews, reporting evidence in 1:3:25 format or policy briefs, empowerment training courses, and some techniques for contextualization including policy dialogue (The Canadian Health Services Research Foundation, 2009; Lavis et al., 2005; Lavis, Boyko, Oxman, Lewin, & Fretheim, 2009; Ward & Mowat, 2012).

Evidence shows that health systems fail to make optimal use of evidence (Hanney, Gonzalez-Block, Buxton, & Kogan, 2003; Innvær, Vist, Trommald, & Oxman, 2002; Lavis et al., 2005; Oxman, Lavis, & Fretheim, 2007). One effort to bridge the gap between knowledge and policy can be undertaken by the policy making organization (pull efforts) (Ellen, Lavis, Ouimet, Grimshaw, & Bédard, 2011). A review of the literature shows that successful implementation of strategies to reduce the know-do gap highly depends on identifying barriers and facilitators in each specific setting (Graham et al., 2006; Graham & Tetroe, 2007; Lavis, 2006).

### 1.2 Area Descriptions

We believe that it is unrealistic to expect policy makers to find and appraise evidence and adapt policies to local conditions. In fact, policy makers are the end-users of the evidence that is synthesized by their consultants or senior civil servants (SCSs).

Promotion of EBPDs that are well developed by SCSs and are timely available can reduce the barriers to evidence utilization by health policy makers. Studies have focused on the factors influencing the use of evidence in health policy. The present study is based onevidence-informed policymaking. This approach considers scientific evidence as only one of the inputs in the policy making process. It focuses on evidence-informed policy rather than evidence-based policy. Therefore, our focus in this study is on policy documents, an objective product, rather than the policy making which is a subjective process. This research was designed to study barriers and facilitators of developing EBPDs from the perspective of their producers in IMoH.

## 2. Methods

**Figure 1:**
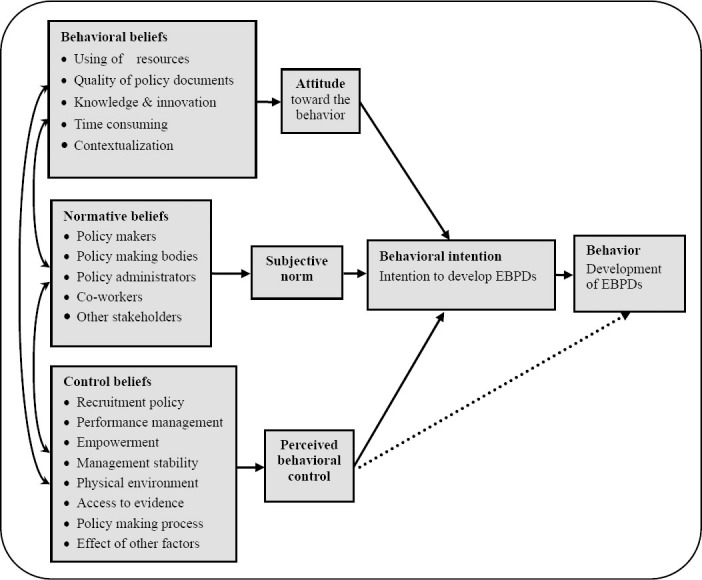
Factors affecting the development of evidence-based policy documents in Iran

A qualitative study with a framework analysis approach was used for data collection and analysis. Studies of the barriers and facilitators of a phenomenon are either exploratory or theory-based (Straus, Tetroe, & Graham, 2013). In this study, the theory of planned behavior (TPB) was used as a framework to identify barriers and facilitators in developing EBPDs. TPB is one of the most famous theories to explain human behavior (Duan & JIANG, 2008). This theory postulates three determinants of intention: attitude toward the behavior, subjective norm, and perceived behavioral control. The intention is a strong predictor of future behavior (Ajzen, 1991) (Figure 1). TPB has proved its power to predict behaviors in different areas, including health care (Eccles et al., 2006). This theory helps us identify the main factors that influence a behavior, while the exploratory approach may overlook some of these factors.

We conducted semi-structured interviews with a purposive sample of EBPDs producers, directors of technical offices (D), and their senior Technicians (T) in IMoH. Those with a minimum of five years’ experience in policy development at the national level were interviewed. Snowball sampling was also used to identify key informants. IMoH consists of nine deputies, each deputy consists of a few bureaus/centers, and each bureau/center consists of several technical offices (IMoH, 2013). Technical offices are the starting point for developing an EBPD in IMoH.

An interview guide, informed by TPB, was developed and pilot-tested with 5 interviewees (Appendix). The interview guide included questions about the advantages and disadvantages of developing EBPDs, individuals or groups that approve or disapprove of EBPDs, and conditions that facilitate or hinder the development of EBPDs.

All interviews were conducted face-to-face by the first author (INMH) from January to February 2013. INMH has 12 years of experience in IHS. There was no communication between the interviewer and the participants prior to the study. To ensure confidentiality, all interviews were performed in the participant’s individual office or in the meeting room with nobody else present. Interviews lasted between 32 and 78 minutes (53 min. on average). Verbal informed consent was obtained before recording the interviews by an electronic voice recorder. The data collection continued until the point of data saturation.

All of the recorded interviews were transcribed and coded based on framework analysis developed by Ritchie and Spencer. The key steps in framework analysis are: familiarization, identifying a thematic framework, indexing, charting, and mapping and interpretation (Ritchie & Spencer, 2002). Based on the aims of the study and familiarization, 18 codes and three themes of behavioral, normative, and control beliefs were identified. To establish inter-coder reliability, two members of the research team coded two interview transcripts and discussed and removed the inconsistencies (Carey, Morgan, & Oxtoby, 1996). Research findings were sent to the participants to get feedback on our interpretations and to ensure that their intentions and views were faithfully presented (Lincoln & Guba, 1985). A qualitative analysis software (MAXQDA-10) was used to apply the codes and manage the data. COREG (consolidated criteria for reporting qualitative research) checklist was used for reporting this study (Tong, Sainsbury, & Craig, 2007).

This study was based on theory and the results were compared to the findings of exploratory studies on the factors that affect evidence utilization by health policy makers. To this end, factors affecting evidence utilization by health policy makers were classified based on the TPB components (Albert et al., 2007; Bowen et al., 2009; Hennink & Stephenson, 2005; Hyder et al., 2011; Orem et al., 2012).

## 3. Results

We interviewed 23 producers of EBPDs in IMoH. Table 1 shows the characteristics of the participants.

**Table 1:** Characteristics of the participants

Characteristics	Experience (yrs.)		Background		Employment Status		Degree			Management Experience	
	5-10	11-16	Clinical	Non-Clinical	Technician	Faculty Member	MS	GP	PhD	Yes	No
Number	13	10	12	11	19	4	8	7	8	10	13

MS: Master of Science; GP: General Practitioner; PhD: Philosophy of Doctor.

Figure 1 presents our finding within the framework of TPB.

### 3.1 Behavioral Beliefs

Behavioral beliefs refer to one’s perception of the consequences of a particular behavior. The positive or negative evaluation of behavioral beliefs determines one’s attitude toward the behavior (Ajzen, 1991) (Figure 1). Most of the participants believed that the development of EBPDs improves the quality of proposed policies. The interviewees believe that evidence can be used to inform all stages of the policy-making process (D_2_& T_2, 7, 14_). They believed that using valid evidence leads to correct identification of policy issues, adoption of the most effective policy options, selection of the best implementation strategy, and proper evaluation of policy effects. “*I think the best way to properly prioritize community health needs is to refer to evidence*” (T_14_).

Some of the directors and the majority of the technicians believed that appropriate use of resources, both in the supply and the demand side, is a positive outcome of EBPDs. They argued that much money is spent on research, and it will be a waste of financial resources if the results are not put to practice (T_4, 11, 15_). Interviewees believed that evidence utilization by policy making organizations results in the optimum use of financial resources in the health sector, especially in developing countries where resources are scarce (D_2, 5, 6_).

Some technicians argued that they have always been concerned about financial losses caused by the proposed policy options to the health system and society (T_1, 2_): “*What will happen if we don’t support health policies by evidence and they are approved and implemented? Many valuable resources are devoted to them which could have been spent on appropriate policies which would work. This results in the waste of my country’s resources*” (T_1_). They also believed that EBPDs improve the opportunity-cost of financial resources in the health system (T_1, 11_). “*Using of evidence leads to adoption of proper policies which won’t need pilot implementation*” (M_5, 6_).

The technicians mentioned that preparation of EBPDs results in the development of their knowledge and innovation (T_2, 3, 4, 5_). They consider it a satisfying and motivating experience (T_1, 9, 10_). Interviewees believed that new learning experiences are the least benefit of evidence review regardless of its use in the policy making process (T_1, 9_).

Most of the participants reported that developing EBPD is a time-consuming process. They believed that this may result in losing the chance to swiftly respond to problems. “*Developing EBPDs is often so slow that we get the feeling that we’re losing opportunities*” (D_1_).

One director and some technicians believed that developing EBPDs creates a tendency toward policies that are supported by international evidence, but may not be adaptable to the Iranian context (D_7_ & T_4, 5, 9_).

### 3.2 Normative Beliefs

Normative beliefs are perceptions of social pressure to perform or avoid a particular behavior. The positive or negative judgments about normative beliefs determine the prevailing subjective norm (Ajzen, 1991) (Figure 1). “*The attitude of policy makers towards evidence depends on the extent to which the issue is political. This means that if they are free of external pressure they will prefer evidence-based policy making*” (T_15_).

Most of the interviewees believed that policy authorities such as the Parliament, the High Council of Health, and the Vice President of Strategy support EBPDs. But sometimes regional interests of some parliamentarians lead to their opposition against evidence that supports national interests (D_1_ & T_3, 13_). At times, non-governmental organizations such as patients associations and professional bodies such as Medical Associations oppose the use of evidence in health policy due to their interests (D_1, 7_ & T_1, 4, 11_). The participants believed that the scientific societies always support EBPDs (T_1, 8, 11_).

Most of the interviewees believed that policy administrators often resist change. They argued that using evidence to support health policies can decrease the resistance (D_2, 5_ & T_3. 8_). The participants believed that those co-workers with EBPD skills support this behavior (T_8_).

### 3.3 Control Beliefs

Control beliefs are people’s perception of their control over their behavior. These beliefs are concerned with the perceived power of specific factors to facilitate or inhibit performance of the behavior (Ajzen, 1991) (Figure 1).

All of the interviewees noted the weaknesses in the performance measurement system and the poor relationship between compensation and performance. “*In our system, there is no process to encourage such behavior; there isn’t a proper performance evaluation process for technicians and technical offices. Our system doesn’t properly compensate the services of its employees*” (D_2_).

Some interviewees believed that appointing faculty members as IMoH policy makers can facilitate the use of evidence in policy development (D_8_ & T_1, 15_). They also noted that skill in the development of EBPDs is not a “must” criterion for employment in IMoH (D_1_ & T_6, 10, 14_).

The participants noted lack of management stability as a barrier to the development of EBPDs (D_1, 4_ & T_1, 7, 8, 11_). “*Policy makers are now supporting this behavior, but there is no guarantee that future policy makers will have the same attitude*” (D_4_).

The majority of interviewees believed that rejection of non-evidence-based policies by senior policy authorities can facilitate the development of EBPDs (T_1, 2, 3, 4, 5_). But they did not consider it a common strategy for all IMoH bodies involved in policy making (D_4, 7_ & T_2_).

Most participants believed that empowering courses and workshops are essential to the development of EBPDs (D_3, 6_ & T_2, 4, 6, 8_), but not sufficient: “*A few evidence-based health policy workshops are held, but they are not practical and useful*” (T_8_). Moreover, the majority of interviewees mention crowded workplace as a barrier to the development of EBPDs (D_2_ & T_4, 5, 8_).

Access to evidence was evaluated from three perspectives: stakeholders’ view, current data on the organization, and research evidence. Lack of facilities such as teleconference and a database to identify researchers leads to inefficient use of researchers’ and stakeholders’ views (D_3, 5, 8_ & T_2, 5, 6, 14_). “*In the implementation of the family physician policy, we didn’t consider the views of providers and receivers of services; so we couldn’t identify the barriers to policy implementation correctly* (E_7_).”

Another obstacle was lack of access to the full text of some articles (T_1, 2, 10_). Some of the directors and technicians considered insufficient access to the current data of health system, especially the cost of services, as a barrier to developing EBPDs (D_2, 8_ & T_1, 3, 10_). “*To measure the cost-effectiveness of an intervention, we can simply use published evidence; but unfortunately, there are no valid data to estimate the cost of interventions*” (D_2_).

Published studies about the factors that influence evidence utilization by health policy makers were exploratory. These studies did not use social cognitive theories as a framework for data collection and analysis. They also did not classify the driving factors as behavioral or subjective beliefs. In other words, all the factors identified in these studies were classified as control beliefs.

## 4. Discussion

This study provides insights about the factors that influence the development of evidence-based policy documents in a developing country. Published studies focused on the factors influencing use of evidence in health policy making. But this study focused on one step before the policy making which is development of policy document in the technical offices in IMoH. This phase is objective and is the end point of evidence-informed health policy making process.

In this study, improving the quality of policy documents, preventing the waste of resources, and developing knowledge and innovation were identified as positive behavioral beliefs, while being time-consuming and lacking adaptability were identified as negative behavioral beliefs. Positive attitude of health policy makers toward the use of evidence in policy making has been reported in some studies (Bowen et al., 2009; Jönsson, Tomson, Jönsson, Kounnavong, & Wahlström, 2007; Peirson, Ciliska, Dobbins, & Mowat, 2012). However, policymakers were concerned about the validity of the evidence (Hennink & Stephenson, 2005; Jönsson et al., 2007; Orem et al., 2012). Although policy makers may have a positive attitude toward evidence, in practice they prefer other factors such as pressure groups to evidence (El-Jardali et al., 2012; Hyder et al., 2011; Orem et al., 2012). Apparently, the reason that producers of policy documents insist on the use of evidence is that they are not under pressure by other factors. Lack of concern about political popularity can also play an important role in this regard. Some studies have also cited the time-consuming nature and contextualization of policies as challenges of evidenced-based policy making (Albert et al., 2007; Campbell et al., 2009; Dobrow et al., 2004).

Most of the interviewees believed policy authorities and scientific associations support EBPDs, while policy administrators, patients associations, and professional bodies tend to resist against evidence utilization in policy documents. The attitude of their co-workers and policy makers depends on the circumstances. Some studies have shown that political actors such as professional bodies do not value the evidence. Resistance of administrators against new policies has also been cited in the some studies (Bowen et al., 2009; Dobbins, Rosenbaum, Plews, Law, & Fysh, 2007; El-Jardali et al., 2012).

Among control beliefs, appointment of faculty members as health policy makers was identified as a facilitator of EBPDs. This is consistent with the results of other studies, as they suggest experience in the use of evidence in policy making as a criterion for appointing health policy makers (Majdzadeh et al., 2012; Orem et al., 2012).

Lack of evidence-based policy making (Dobbins et al., 2007; Majdzadeh et al., 2012), rapid replacement of policy makers (Majdzadeh et al., 2012; Orem et al., 2012), poor knowledge and skills of policymakers in evidence utilization, and limited access to required evidence have also been reported as important barriers in the most studies related to the use of evidence by health policy makers (El-Jardali et al., 2012; Ellen et al., 2013; Koehlmoos, Rashid, Rahman, Cravioto, & Hanney, 2009; Majdzadeh et al., 2012).

Table 2 shows specific factors that affect the development of EBPDs as well as evidence utilization by heath policy makers. In this table, common factors affecting both the development of EBPDs and evidence utilization by health policy makers were excluded.

**Table 2:** The differences between the factors affecting the development of EBPDs and evidence utilization by health policy makers

Factors affecting the evidence utilization by policy producers	Factors affecting evidence utilization by heath policy makers
• Development of knowledge and innovation	• Use of jargon
	• Evidence structure
• Performance management	• Time constraint
• Physical environment	• Timely and relevant evidence
• Recruitment of technicians	• Administrative structure to support evidence-based policy making
• Co-workers	• Limited resources to support evidence-based policy making

One of the most popular frameworks for identifying the barriers to implementation of new interventions is developed by Hanson et al (Hanson, Ranson, Oliveira - Cruz, & Mills, 2003). In this framework, the barriers to a change are identified in four levels—providers and recipients of services, organization, and the health system. Our findings covered all the components of this framework based on the theory of planned behavior (TPB) (see table 3). While the barriers identified in the exploratory studies on use of evidence by health policy makers were mostly related to the organization and health system levels, a few studies have reported barriers at the provider or recipient levels (Albert et al., 2007; Bowen et al., 2009; Hennink & Stephenson, 2005; Hyder et al., 2011; Orem et al., 2012). This finding poses the question whether using TPB as a framework for data gathering and analysis can help to identify the barriers to a particular behavior at all the levels of the health system.

**Table 3:** Factors affecting the development of EBPDs in the framework of Hanson et al. (2003)

Components of the framework	Findings of our study
Service providers	• Attitude and skills of the producers of EBPDs (behavioral beliefs+ skill)
Service recipients	• Attitude of health policy stakeholders (normative beliefs)
Organization	• Performance management, empowerment, physical environment, and technicians recruitment (control beliefs)
Health system	• Management stability, access to evidence, appointment of policy makers, and other factors (control beliefs)

We found that most of the barriers to develop EBPDs were control beliefs. The results of other studies support this finding, as they imply that the real challenges of research utilization in health policy are structural, environment, or system barriers (Bowen et al., 2009; Majdzadeh et al., 2012).

The factors identified in the studies on evidence utilization by health policy makers were classified as control beliefs. This finding implies one of the following hypotheses: (1) factors related to other components of TPB do not affect evidence utilization by health policy makers; (2) by not using TPB, these studies tend to neglect factors related to other components; or (3) the most important factors influencing evidence utilization by health policy makers and development of EBPDs are related to control beliefs. So other identified factors in this study do not have significant effects, but asking about them in the interviews resulted in talking about them.

Our findings cannot be generalized to other settings because of its design. Although lack of generalizability is the major limitation of qualitative studies, they can provide important insights into the factors that affect a phenomenon through in-depth interviews with key informants (Holloway, 2005).

## 5. Conclusion

In spite of the participants’ concerns about time-consuming and contextualization, their attitude toward developing EBPDs was positive. We showed that not all the stakeholders of health policies support EBPDs. The main barriers to the development of EBPDs were related to control beliefs (barriers related to the organization and health system levels in Hanson’s framework). This finding remind us of the 85:15 rule in Total Quality Management (TQM), which claims that 85% of quality problems are related to processes and systems, while employees may cause only 15% of quality problems (Deming, 1986). Our theory-based study identified the factors that influence the development of EBPDs in terms of all the components of TPB, while the factors identified the exploratory studies evidence utilization by health policy makers are only related to one of these components. This can suggest that theoretical approaches are preferable to exploratory ones in examining the factors that affect a behavior.

## Appendix

***Interview guide: affecting factors on the development of evidence-based health policy briefs?***

- What do you believe are the *advantages* development of evidence-based health policy documents?
- What do you believe are the *disadvantages* of development of evidence-based health policy documents?
- Is there anything else you associate with your own views about development of evidence-based health policy documents?
- Are there any individual or groups who would *approve* of your development of evidence-based health policy documents?
- Are there any individual or groups who would *disapprove* of your development of evidence-based health policy documents?
- Is there anything else you associate with other people’s views development of evidence-based health policy documents?
- What factors or circumstances would enable you to development of evidence-based health policy documents?
- What factors or circumstances would make it difficult or impossible for you to development of evidence-based health policy documents?
- Are there any other issues that come to mind when you think development of evidence-based health policy documents?
